# 3D FRP Reinforcement Systems for Concrete Beams: Innovation towards High Performance Concrete Structures

**DOI:** 10.3390/ma17122826

**Published:** 2024-06-10

**Authors:** Handong Yan, Jiabao Zhao, Jianli Yin, Wei Sun

**Affiliations:** 1 College of Civil Engineering, Huaqiao University, Xiamen 361021, China; 2 Higher-educational Engineering Research Centre for Intelligence and Automation in Construction of Fujian Province, College of Civil Engineering, Huaqiao University, Xiamen 361021, China; 3 Key Laboratory for Intelligent Infrastructure and Monitoring of Fujian Province, College of Civil Engineering, Huaqiao University, Xiamen 361021, China; 4 KZJ New Materials Group Co., Ltd., Xiamen 361101, China; kzj30@lets.com

**Keywords:** CFRP rupture, load-deflection stiffness, pseudo-ductile behavior, aggregate coating, concrete cracking, apparent CFRP modulus, concrete

## Abstract

Despite the advantages of using lightweight and non-corrosive carbon fiber reinforced polymer (CFRP) reinforcements in concrete structures, their widespread adoption has been limited due to concerns regarding the brittle failure of CFRP rupture and its relatively softer load-deflection stiffness. This work offers logical solutions to these two crucial problems: using aggregate coating to strengthen the CFRP-concrete bond and ultimately the load-deflection stiffness, and using CFRP-concrete debonding propagation to create pseudo-ductile behavior. Subsequently, the concrete cracking behavior, the apparent CFRP modulus with aggregates, and the post-peak capacity and deflection of three-dimensional (3D) CFRP-reinforced concrete are all described by equations derived from experiments. These formulas will be helpful in the future design of non-prismatic concrete components for low-impact building projects. The potential of this innovative design scheme in terms of increased capacity and deflections with less concrete material is demonstrated through comparisons between non-prismatic CFRP-reinforced concrete and measured steel reinforced equivalency.

## 1. Introduction

Fiber reinforced polymer (FRP) composites have the potential to be highly effective reinforcements in concrete beams because of their lightweight nature, high strength, and little or no maintenance requirements due to their non-corrosive properties [1,2,3]. Nonetheless, one- or two-dimension (1D or 2D) FRP reinforcement designs for concrete structures are currently employed as direct substitutes for steel reinforcements in the form of the tendon [4,5], rebar [6,7], grid [8], rope [9], tube [10], plate and sheet [11,12]. These modern systems exhibit intrinsic weaknesses including poor stiffness, brittle failure, insufficient deformation, and a weaker bonding with concrete than with steel [13,14,15]. Meanwhile, modifications have been made to incorporate FRPs into the traditional steel-reinforced concrete design concept [16,17]. However, these modifications further compromise the structural efficiency of FRP-reinforced concrete.

Although as a means of preventing the brittle failure of FRP rupture in reinforced designs are suggested, this generally results in another undesirable failure of concrete crushing. Ideally, FRP-reinforced concrete should gradually develop considerable deformation up to the desired mode of ultimate failure. Thus, efforts were undertaken to investigate more sophisticated FRP reinforcement systems composed of multidirectional fibers in order to achieve the desired levels of ductility and deformation [2,18,19,20]. For example, in the FRP system, which has both 0° and ±45° fibers, the ±45° fibers will help to transfer the load when the 0° fibers fail at the corresponding tensile strength of the material. This characteristic guarantees a progressive decrease in the load capacity following the peak load. This pseudo-ductility could be developed for FRP-reinforced concrete by exerting a controlled FRP-concrete debonding [21,22,23,24,25]. Then, anchorage systems could be provided for obtaining additional capacity and preventing prematurely debonding failure [26,27]. 

Because FRPs have a relatively lower Young’s modulus than steel, concrete beams will have less flexural stiffness than equivalent steel-reinforced beams. Nevertheless, compared to an analogous beam strengthened with steel bar reinforcements, the adoption of 3D forms of the reinforcement will assure higher tension-stiffening effects [2] and smaller and fewer flexural cracks [28,29]. Furthermore, aggregate coatings on FRPs provide additional stiffness, strengthening the bond between the FRP and the concrete and expanding the nominal area of FRP composites [30,31]. Therefore, it is expected that beams can attain greater flexural stiffness when reinforced with alternative types of 3D FRP reinforcement in comparison to beams reinforced with traditional FRP bars.

Prior to curing with epoxy, the flexible FRP fabric could be effectively manipulated to create an innovative and effective three-dimensional (3D) system that included both shear and flexural reinforcement. This system is designed to fulfill both capacity and serviceability criteria [2]. With a proper design, this adaptable technology can be utilized as a stay-in-place formwork in the future to cast concrete with complex geometries, potentially saving a significant amount of embodied energy and building materials [32,33]. In light of the concepts, CFRP fabrics (i.e., U-channel (UC), U-channel with an aggregate coating (UCA), U-channel with intermittent closed loops (UCL), and U-channel with an aggregate coating and intermittent closed loops (UCAL)) have been developed as novel reinforcements for concrete elements. While experimental results show good displacement ductility and capacity [2], these characteristics cannot be adequately represented by current standards of practice when using less accurate concrete cracking models, CFRP modulus, and a CFRP–concrete bond [16,17].

In addition to the experimental advancements, numerical modeling plays a crucial role in understanding the complex interaction between FRP reinforcements and concrete. These models [14,15,34,35] often consider the material properties of both the FRP and the concrete, the bond characteristics at the interface, and the overall structural geometry to simulate the behavior accurately.

However, the models generally focus on analysis rather than extending to the design phase, where they assist in optimizing the use of FRP reinforcement. This study aims to develop the concrete cracking model, in situ CFRP modulus, and CFRP-concrete bond for CFRP-reinforced concrete beams, drawing upon experimental findings. To investigate the CFRP-reinforced concrete beams’ pre-peak and post-peak behavior, those calibrated models will be integrated with a MATLAB code [36]. Subsequently, the updated script will consider the intricate geometries of 3D concrete elements in order to create an optimal design that will be contrasted with comparable research on low-impact concrete elements [32]. The key to an optimal design is accurately capturing the combined effects of post-peak behaviors, enhanced/compromised FRPs, and cracked concrete, all of which have not received enough attention in previous research but will be discussed in this study [36].

## 2. Beam Test Specimens

**Figure 1:**
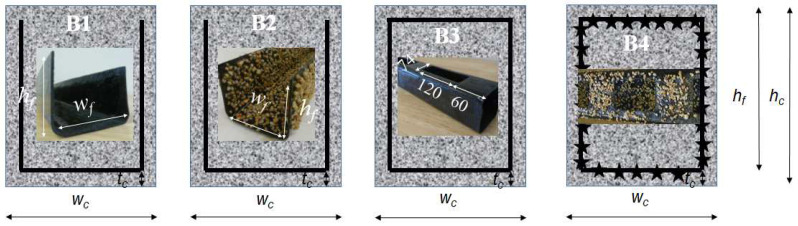
Test specimens [2].

**Figure 2:**
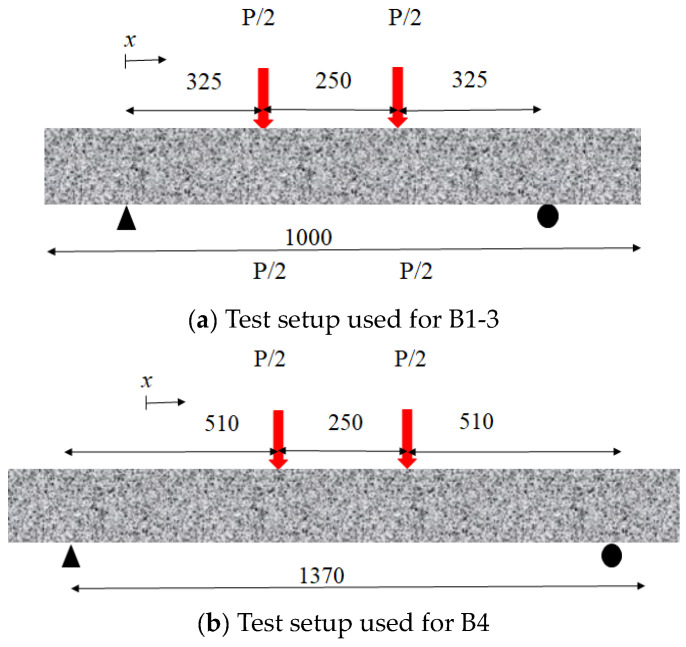
Four-point bending test.

Four-point bending tests were used to illustrate the loading behavior of four beam specimens with various 3D CFRP reinforcements, i.e., UC, UCA, UCL, and UCAL [2] (see Figure 1). The first three beams (i.e., B1-3) had a constant dimension of 1000 mm (length *L*) × 120 mm (width *w_c_*) × 120 mm (height *h_c_*) with a 20-mm concrete cover *t_c_*, 80-mm-wide CFRP channel (*w_f_*) and a 100-mm CFRP depth *h_f_*, which results in a 325-mm shear span *L_s_* and a 250-mm moment span *L_m_* (see Figure 2a). It should be noted that small aggregates (up to 10 mm) were selected to facilitate the casting process. The apparent FRP modulus would be proposed to capture the tensile improvement brought by the aggregate. The 3D CFRP reinforcements of the first three beams were UC/B1, UCA/B2, and UCL/B3, respectively. The fourth beam specimen UCAL/B4 had a unique size of 1370 mm × 150 mm × 100 mm with a 510-mm shear span, a 250-mm moment span, a 110-mm-wide CFRP channel (*w_f_*), a 80-mm CFRP depth *h_f_*, and a 20-mm concrete cover [37]. The concrete strength was 38 MPa for B1-3 and 25 MPa for B4. Four identical CFRP strips with ±45° fiber were applied to fabricate all CFRP reinforcements resulting in a nominal 1.65-mm thickness, a 23.6-GPa modulus and a 457-MPa tensile strength. Aggregates of the size 2–5 mm were selected for UCA and UCAL reinforcements applied in specimen B2 and 4, respectively. All four tests developed considerable deflections after the peak loads, and eventually failed at a compressive strain of concrete *ε*_c_top_ of 0.0019 for B1, 0.0026 for B2, and 0.003 for B3 and 4.

The concrete beams were cast and tested at the age of 34 days, which is a common practice to allow the concrete to reach its full compressive strength. The loading speed for the four-point bending tests was a ramped loading at a stroke rate of 2 mm/min. This rate is chosen to ensure a controlled and consistent application of force throughout the test. Various types of sensors were used to collect data during the tests. Strain gauges were applied to the CFRP reinforcement channels and to the top and side surfaces of the concrete beams to measure strains. These strain gauges were connected to a StrainSmart data logger for recording the data. The use of these sensors provided a comprehensive set of data for analyzing the performance of the FRP-reinforced concrete beams. More details about the four specimens can be found in the research work [2].

## 3. Concrete Cracking Model

**Figure 3:**
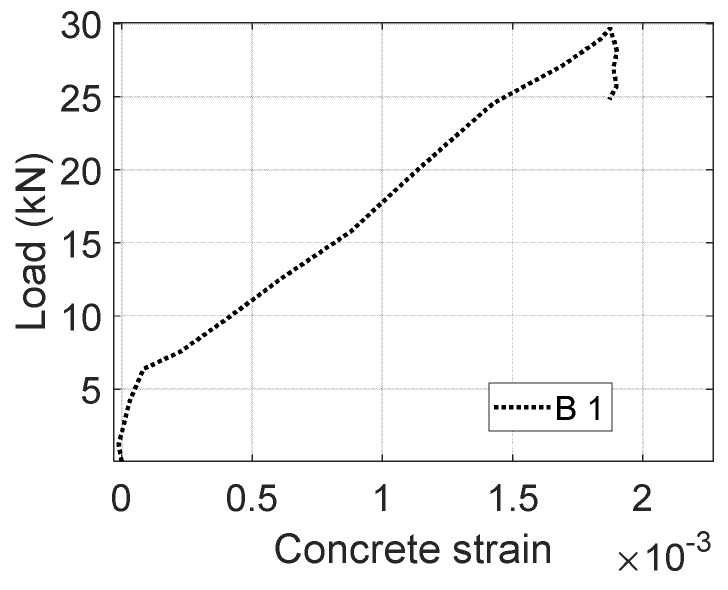
Load versus concrete strain of B1.

**Figure 4:**
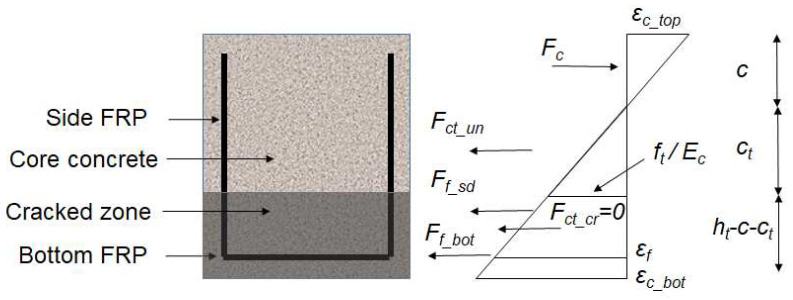
Strain distribution and resultant forces.

The measured load versus concrete strain (see Figure 3) of B1 was applied to develop a concrete cracking model. As shown in Figure 4, CFRPs tend to develop less strain deformations as the location is approaching the neutral axis. The side CFRPs are therefore able to act as anchorages to limit the CFRP–concrete slip at the bottom. A no-slip assumption has been made to describe the CFRP–concrete bond of B1 before the peak load is reached. Moreover, the contribution of cracked concrete is initially assumed to be zero after the tensile strength of concrete is reached. The equilibrium can be presented as follows:

(1) $$F_{c}-F_{ct\_un}-F_{ct\_cr}-F_{f\_sd}-F_{f\_bot}=0$$

(2) $$M_{c}+M_{ct\_un}+M_{ct\_cr}+M_{f\_sd}+M_{f\_bot}-P/2\times L_{s}=0$$

in which

$F_{c}$ and $M_{c}$ are the compression force of the concrete and the resultant moment;

$F_{ct\_un}$ and $M_{ct\_un}$ are the tensile force of the uncracked concrete and the resultant moment;

$F_{ct\_cr}$ and $M_{ct\_cr}$ are the tensile force of the cracked concrete and the resultant moment;

$F_{f\_sd}$ and $M_{f\_sd}$ are the tensile force of the side CFRPs and the resultant moment;

$F_{f\_bot}$ and $M_{f\_bot}$ are the tensile force of the bottom CFRP and the resultant moment;

P is the applied load as shown in Figure 2.

The compression force $F_{c}$ is obtained from concrete stress $f_{c}$ which can be determined by inputting the concrete strain $\varepsilon_{c}$ into the following equations [38]:

(3) $$f_{c}=f_{c}^{\prime }\left[2\varepsilon_{c}/\varepsilon_{0}-{\left(\varepsilon_{c}/\varepsilon_{0}\right)}^{2}\right]$$

(4) $$\varepsilon_{0}=1.8f_{c}^{\prime }/E_{c}$$

(5) $$f_{t}=\varepsilon_{t}E_{c}$$

in which

**Figure 5:**
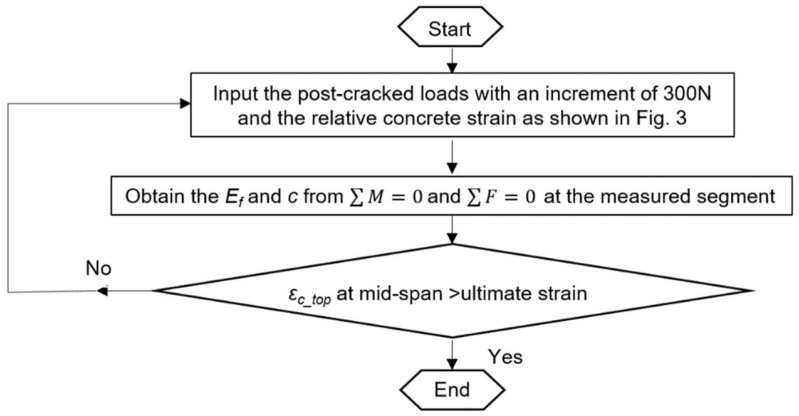
Step-by-step procedure to determine *E_f_* and *c*.

$f_{c}^{\prime }$ and $E_{c}$ are the concrete modulus and compressive strength, respectively;

$f_{t}$ and $\varepsilon_{t}$ are the concrete tensile strength and relative strain, respectively;

Then, the compressive depth *c* and the CFRP modulus *E_f_* can be determined by inputting Load *P* versus the concrete strain into the following equations (see Figure 5):

(6) $$F_{c}=w_{c}cf_{c}^{\prime }\left[\varepsilon_{c\_top}/\varepsilon_{0}-{\left(\varepsilon_{c\_top}/\varepsilon_{0}\right)}^{2}/3\right]$$

(7) $$M_{c}=w_{c}c^{2}f_{c}^{\prime }\left[{2\varepsilon }_{c\_top}/3\varepsilon_{0}-{\left(\varepsilon_{c\_top}/\varepsilon_{0}\right)}^{2}/4\right]$$

(8) $$F_{ct\_un}=w_{c}f_{t}c_{t}/2$$

(9) $$M_{ct\_un}=w_{c}f_{t}c_{t}^{2}/3$$

(10) $$F_{ct\_cr}=\left(h_{t}-c-c_{t}\right)w_{c}f_{t}\alpha_{t}$$

(11) $$M_{ct\_cr}=F_{ct\_cr}\left[\left(h_{t}-c-c_{t}\right)/2+c_{t}\right]$$

(12) $$F_{f\_sd}=\varepsilon_{f}\left(h_{f}-c\right)E_{f}t_{f}$$

(13) $$M_{f\_sd}=2F_{f\_sd}\left(h_{f}-c\right)/3$$

(14) $$F_{f\_bot}=\varepsilon_{f}E_{f}t_{f}w_{f}$$

(15) $$M_{f\_bot}=F_{f\_bot}\left(h_{f}-c\right)$$

(16) $$\varepsilon_{f}/\left(h_{f}-c\right)=\varepsilon_{c\_top}/c=\varepsilon_{c\_bot}/\left(h_{t}-c\right)$$

in which

$\alpha_{t}$ is the factor to describe the reduced strength of cracked concrete;

$\varepsilon_{c\_top}$ and $\varepsilon_{c\_bot}$ are the strains developed at the top and bottom layer of the specimens, respectively;

$\varepsilon_{f}$ is the strain of the bottom CFRP.

It should be noted that few studies have been conducted on the cracking model of 3D CFRP-reinforced concrete. The provision of continual CFRP fabrics is expected to limit concrete cracking which might improve the efficiency of CFRP reinforcement in transferring the tensile force. Nevertheless, current rules of practice, which typically assume that cracked concrete does not contribute to tension, do not adequately characterize the improvement (i.e., $\alpha_{t}=0$). Thus, tension forces are solely carried by CFRPs. As shown in Figure 4, the bottom CFRP is parallel to the longitudinal direction of the cracked zone. After flexural cracking, it was observed that concrete fragments adhered to the CFRP surface, aiding in transferring the tensile force. This observation, generally neglected in conventional design procedures, serves to improve the apparent stiffness of CFRPs. In this study, the stiffness improvements can be demonstrated by the apparent modulus of the bottom CFRPs.

**Figure 6:**
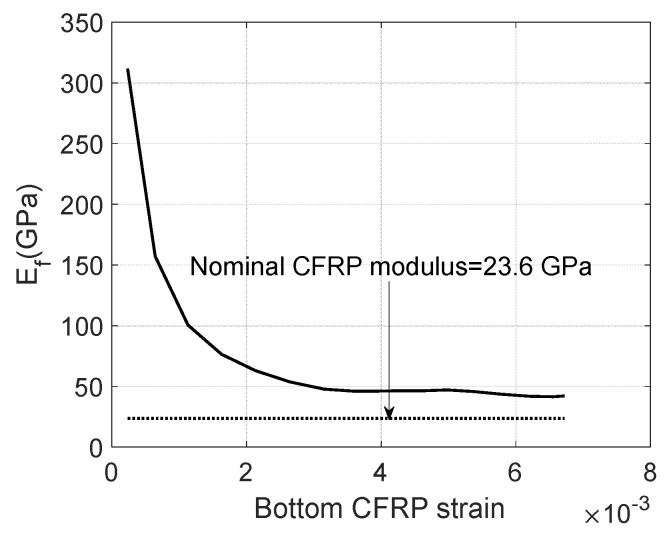
Bottom CFRP strain versus *E_f_*.

A nominal modulus of 23.6 GPa is applied for side CFRPs because few additional improvements can be obtained from their bonds with uncracked concrete and the same cracked zone as the bottom CFRPs as shown in Figure 4. By using Equations (1)–(16) as shown in Figure 5, the apparent modulus of bottom CFRPs is presented in Figure 6. In order to minimize the noise during small measurements, only post-cracked strains have been used to determine the CFRP modulus. As shown in Figure 6, the required *E_f_* of the bottom CFRPs has to be at least twice as large as the nominal value to carry the tensile force. This suggests considerable tension contributions of cracked concrete which might partially bond on CFRP to improve the stiffness of CFRPs until the ultimate failure. The contribution of cracked concrete can be presented by using a reduced factor $\alpha_{t}$ as follows:

(17) $$\alpha_{t}=\left(E_{f}-nominalE_{f}\right)\varepsilon_{f}A_{f}/\left(w_{c}f_{t}\left(h_{t}-c-c_{t}\right)\right)$$

in which $A_{f}$ is the area of the bottom CFRPs.

**Figure 7:**
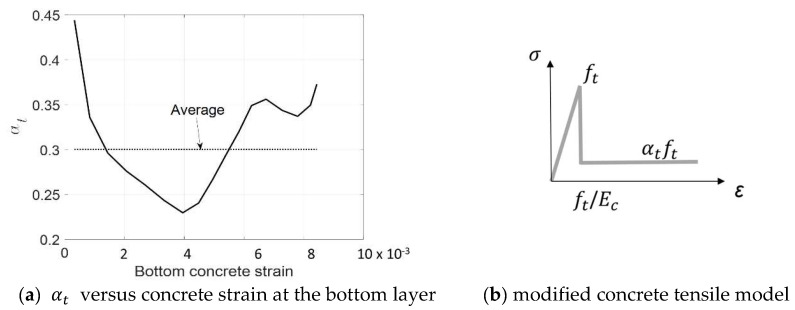
Tensile behavior of cracked concrete.

Figure 7a shows the relationship between $\alpha_{t}$ and the tensile strain of concrete with a limited range not exceeding 0.0085. The value of $\alpha_{t}$ varies randomly around an average value of 0.3 while the nominal strain at the bottom layer of the concrete increases. A reduced factor $\alpha_{t}$ of 0.3 is therefore applied in the following calculations to describe the tensile contribution of cracked concrete. The modified tensile model is shown in Figure 7b. It should be noted that the trilinear strain-hardening tensile models with reduced factors ranging from 0.3 to 0.35 were developed from the direct tensile tests on FRP textile reinforcement concrete specimens [28,29]. In this study, a more suitable value of 0.3 is suggested for the flexural design of the 3D CFRP-reinforced concrete beams.

## 4. Effective Modulus for U-Channel CFRP Reinforcements

The experimental results found that the usage of a size 2–5 mm aggregate coating U-channel (UCA) tended to improve the CFRP–concrete bond [2]. Thus, a very thin layer of concrete might well bond with CFRPs to help transfer the tensile force till the ultimate failure, which would increase the apparent stiffness of CFRPs. On the other hand, the close loops (UCLs) prevented fresh concrete from contacting well with CFRPs during the casting [2]. This unfavorable contacting issue would result in a premature CFRP–concrete slip, compromising the apparent stiffness of CFRPs.

**Figure 8:**
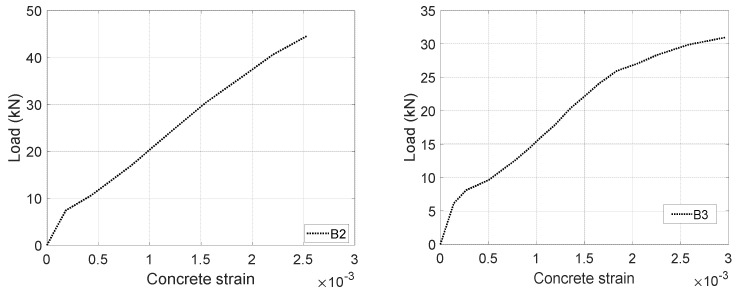
Load versus concrete strain of B2 and B3 [2].

**Figure 9:**
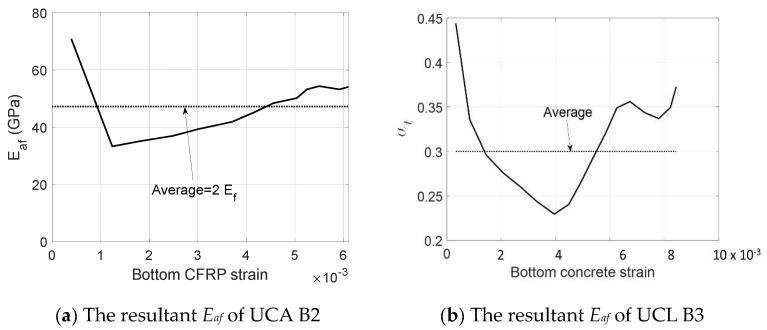
The resultant *E_af_* of B2 and B3.

In this section, the modified CFRP modulus *E_af_* will be applied to capture the variation of the apparent stiffness of CFRPs due to different configurations (e.g., UCA, and UCL). The CFRP modulus *E_af_* is calibrated by inputting the measured load versus concrete strain responses (see Figure 8) into the aforementioned equilibrium and compatibility equations (see Figure 5) with a modified concrete cracking model (see Figure 7b). A reduced factor $\alpha_{t}$ of 0.3 is applied to the cracking model. Then, the resultant CFRP modulus *E_af_* of UCA reinforcements is found as a multilinear curvature around an average value of 2*E_f_*, and the responses of UCL reinforcements are a multilinear curvature around an average value of 0.9*E_f_* (see Figure 9). Thus, the modified CFRP modulus *E_af_* is recommended as 2*E_f_* for UCA and 0.9*E_f_* for the UCL, respectively.

## 5. Post-Peak Calculations

The pre-peak behaviors of 3D CFRP-reinforced concrete (i.e., B1 and B3) have been well described by the equations developed in the literature [36]. The peak has been defined as the compressive strain of concrete that reaches the observed equivalence at the maximum load. In this study, additional equations will be developed to describe the pseudo-ductile behavior of 3D CFRP-reinforced concrete after the peak.

After the peak, the failure of anchorages resulted in a sudden drop in the applied load, and then a plateau up to the ultimate failure [2]. This observation might suggest a debonding propagation of CFRPs after the peak. Then, the post-peak capacity can be determined by the transferred force in the bottom CFRP $F_{f\_bot}$ (i.e., the bond strength $F_{b}$) using the following equation [15]:

(18) $$F_{b}=F_{f\_bot}=w_{f}\sqrt{E_{f}t_{f}\tau_{max}s_{max}}$$

in which

(19) $$\tau_{max}=1.35+0.25\beta_{w}f_{t}+0.62f_{t}$$

(20) $$s_{max}=-0.06+\left(0.88-0.23\beta_{w}^{2}\right)f_{t}^{-0.5}\beta_{w}^{0.5}$$

(21) $$\beta_{w}=\sqrt{\frac{1.9-w_{f}/w_{c}}{0.9+w_{f}/w_{c}}}$$

**Figure 10:**
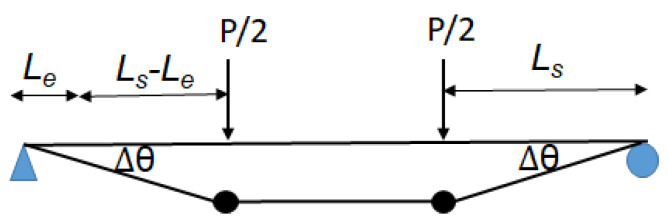
Plastic deflections of the beams.

After the peak load, CFRP–concrete debonding is assumed to occur and propagate from the loaded point to the support points. The debonding propagation is able to produce a CFRP–concrete slip which will allow a rotation around the support points. As shown in Figure 10, two plastic hinges are therefore assumed to be developed after the peak load. This introduces additional rotation Δ*θ* which is the result of a CFRP–concrete slip and the elongation of debonded CFRPs. The ultimate failure occurs when the remaining length of the CFRP–concrete bond is less than the length required to develop the bond strength $L_{e}$ [15]. Therefore, the ultimate deflection ${∆}_{ult}$ can be presented as follows:

(22) $${∆}_{ult}={∆}_{peak}+∆\theta \left({L_{s}-L}_{e}\right)$$

in which

${∆}_{peak}$ is the deflection at the peak which can be determined by using the equations in the literature [36];

∆θ is the rotation producing the plastic deflection as shown in Figure 10;

$L_{s}$ is the distance between the applied load and the support as shown in Figure 10.

(23) $$∆\theta =\left(s_{max}+F_{b}\left({L_{s}-L}_{e}\right)/E_{f}/A_{f}\right)/\left(h_{t}-t_{c}\right)$$

(24) $$L_{e}=1.3\sqrt{\frac{t_{f}E_{f}}{{\left(f_{c}^{\prime }\right)}^{0.58}}}$$

**Figure 11:**
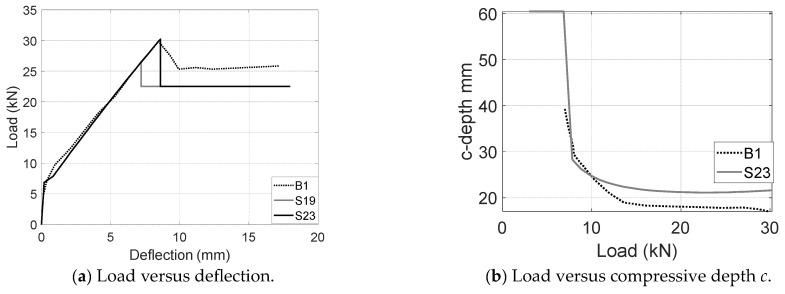
Comparisons between measurements and predictions for B1 (UC).

**Figure 12:**
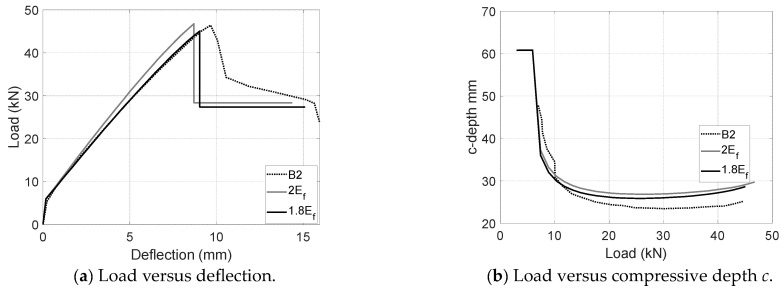
Comparisons between measurements and predictions for B2 (UCA).

**Figure 13:**
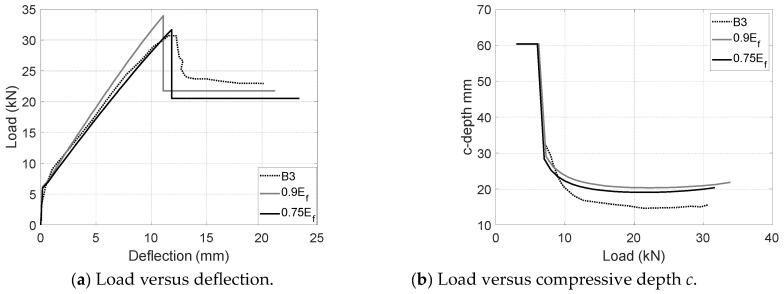
Comparisons between measurements and predictions for B3 (UCL).

As shown in Figure 11a, the observed displacement ductility after the peak agrees well with the assumption of debonding propagation and plastic hinges. By incorporating the proposed concrete cracking model, the post-peak capacity, and the ultimate deflection with the deflection, equilibrium, and compatibility equations in the literature [36], the load-versus-deflection responses of B1 can be determined (see Figure 11a). Two calculations (S23 and S19) are presented to demonstrate the impacts of maximum compressive strain which has been applied to determine the peak load. S19 uses the measured value of 0.0019 to develop a peak load slightly less than the measured load. In order to match with the peak load, the maximum compressive strain is expected to be 0.0023 as the prediction of S23. The difference between the measured and required value might come from the limits in usage of local measurements to capture the global compressive behavior. For example, construction, material, and measurement variabilities would produce notable differences between local and global readings. For those comparisons between B1 and S23, the predicted values deliver 101% peak load, 89% post-peak load and 104% ultimate deflections. Moreover, the depth of the compressive zone (around an 18-mm measurement) is also well captured by S23 (21 mm) as shown in Figure 11b. During the elastic stage, the depth approached approximately half of the prism’s total depth. After concrete cracking, a sudden reduction in depth facilitates the quick restoration of the force equilibrium between the FRP and concrete. Subsequent increases in the load result in minimal decreases in the depth as shown in Figure 11b, Figure 12b and Figure 13b.

Load-deflection and load-*c* plots have also been developed for UCA and UCL configurations upon the modified CFRP modulus and the proposed cracking model as shown in Figure 12 and Figure 13. For the aggregate coating configuration (UCA), the modified CFRP modulus is supposed to be twice as large as the nominal modulus. The peak was assumed at the point when the concrete strain reached 0.0032 instead of the measured value of 0.0026. As shown in Figure 12, the prediction in usage of the proposed 2*E_f_* agrees well the load-deflection and load-*c* measurements. A better match can be achieved by slightly reducing the modulus to 1.8*E_f_* which delivers a 97% peak load, 97% post-peak load, 97% ultimate deflections and 114% compressive depth *c*. Inherent differences between local measurements and global behavior might result in a slight variation. Similarly, although the usage of the calibrated modulus 2*E_f_* and the measured compressive strain of 0.003 agree well with the experimental results of UCL specimens (B3), a better agreement can be realized by reducing the modulus from 0.9*E_f_* to 0.75*E_f_* (see Figure 13). The predictions upon the reduced modulus 0.75*E_f_* deliver 103% peak load, around 90% post-peak load, 116% ultimate deflections and 130% compressive depth *c*. It should be noted that the apparent CFRP modulus of UCL configurations largely depends on the construction quality and in situ bond condition, which might result in a relatively larger variation than that of comparable CFRP configurations (e.g., UC and UCA) with an open top helping to achieve a better CFRP–concrete bond.

## 6. Predicted *E_f_* for U-Channel Reinforcements with an Aggregate Coating and Intermittent Closed Loops (UCALs)

Specimen B4 was conducted to demonstrate the performance of UCAL reinforcements consisting of both an aggregate coating U-channel and intermittent closed loops. The ultimate compressive strain is expected to be in a range from 0.003 to 0.0032 based on the experimental results of UCA and UCL tests. In this section, a conservative value of 0.003 was used to deal with a possibly compromised CFRP–concrete bond due to the usage of close loops. Then, three calculations in the usage of upper-bound, best-fit and low-bound CFRP modulus were conducted to predict the local and global behavior of the 3D CFRP-reinforced concrete beams. The upper-bound modulus is the product of the calibrated UCA modulus 2*E_f_* and the calibrated UCL modulus 0.9*E_f_*, i.e., 1.8*E_f_*. The best-fit modulus is the product of the best-matched UCA modulus 1.8*E_f_* and the best-matched UCL modulus 0.75*E_f_*, i.e., 1.36*E_f_*. The lower-bound modulus comes from the calibrated UCL modulus 0.9*E_f_*.

**Figure 14:**
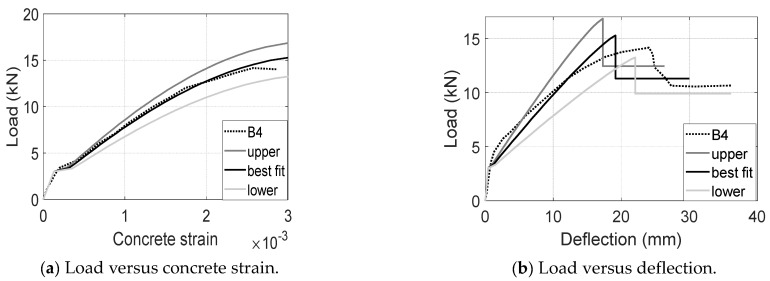
Comparisons between measurements and predictions for B4 (UCL).

As shown in Figure 14a, the load versus concrete strain measurements are within a reasonable range circled by the upper and lower bounds. The calculation in the usage of the best-fit modulus agrees the best with the experimental measurements until the peak load is reached. Then, a limited strain was developed without a further increase in the applied load. This could be the result of CFRP-concrete debonding. Globally, the load-deflection measurements are also within a reasonable range bounded by calculations, in which the one using the best-fit CFRP modulus achieved 108% peak load, 106% post-peak load, and 83% ultimate deflection as shown in Figure 14b. The multilinear pre-peak load-deflection responses might suggest a notable premature CFRP–concrete slip, which reduces the stiffness and capacity of the 3D CFRP reinforcements. Since the pre-mature CFRP–concrete slip greatly depends on construction quality and material variability, the predicted deflection is relatively more conservative than the measured equivalence. In summary, although the best-fit modulus might align closely with the experimental data and provide a more accurate prediction, the lower range is likely to be the safer choice for design purposes.

In this paper, a numerical computational method was developed to demonstrate the potential of four CFRP reinforcements (i.e., UC, UCA, UCL, and UCAL). UC CFRP reinforcements were able to achieve a higher CFRP–concrete bond which resulted in stiff load-deflection responses. However, the beam specimens using UC reinforcements might prematurely fail at a concrete compressive strain of 0.0023 or less. In order to prevent the premature failure, aggregate-coating and close-loop improvements were developed. By coating the aggregate on the surface of UC reinforcements (i.e., UCA), beam specimens are expected to develop stiffer load-deflection responses and fail at a desired compressive strain of 0.003 or larger. Although the usage of close loops is able to increase the ultimate concrete strain up to 0.003, the beam specimen using close-loop schemes (e.g., UCL and UCAL) generally has a compromised CFRP–concrete bond and load-deflection responses which were not readily captured by numerical calculations. Therefore, UCA reinforcements are selected to demonstrate the potential performance of 3D CFRP-reinforced concrete in this section.

**Figure 15:**
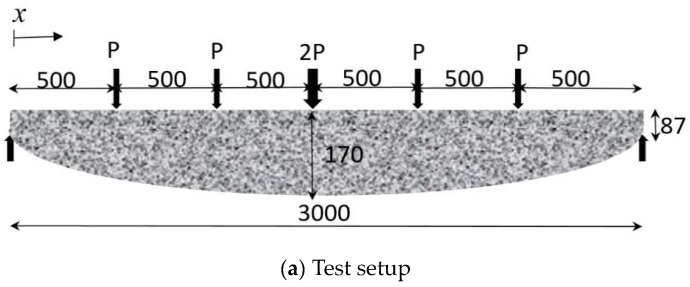

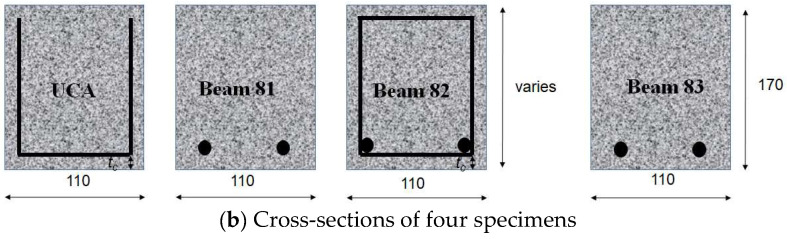
Non-prismatic and prismatic beams for flexural tests.

The UCA reinforcements are fabricated using the same CFRP and aggregate materials as specimen B2, resulting in a nominal thickness of 1.65-mm laminates and a 457-MPa tensile strength. The apparent CFRP modulus is assumed to range from 1.8 *E_f_* to 2 *E_f_*, where *E_f_* is the nominal CFRP modulus of 23.6 GPa. Based on the concrete strength of 38 MPa, a conservative compressive strain of 0.003 is recommended to determine the ultimate failure for this design. Then, the UCA reinforcements will be applied for a non-prismatic beam under a flexural test as shown in Figure 15a. The test setup was developed to determine the behavior of non-prismatic steel reinforced beams (i.e., Beam 81 and 82) and the prismatic equivalence (i.e., Beam 83) as shown in Figure 15b [38]. The non-prismatic geometries of UCA reinforcements are designed according to the moment distribution, allowing for depth variation along the beam’s length as follows:

(25) $$varies=0.0012x-4\times 10^{-7}x^{2}(mm)$$

**Figure 16:**
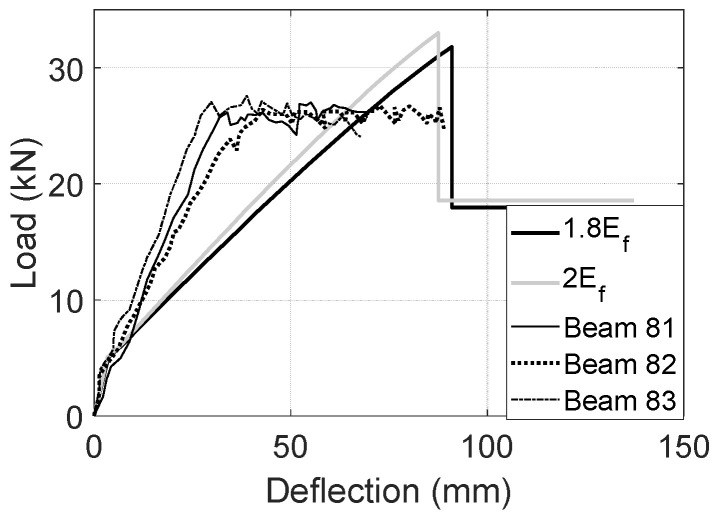
Experimental results of Beam 81–83 [38] and the numerical predictions.

The depth variations of Beam 81 and 82 and other details of the non-prismatic and prismatic tests can be found in the literature [38]. By using the proposed UCA reinforcements and the relative design methods, the predictions in usage of 1.8*E_f_* and 2*E_f_* develop notably larger capacities and deflections than those of comparable steel-reinforced beams, as shown in Figure 16 [38]. Nevertheless, UCA reinforcements tend to result in soft load-deflection responses and lower post-peak loads. In a future study, the load-deflection stiffness could be improved by increasing the thickness of the CFRP laminate. Moreover, higher-strength concrete could be used to improve the CFRP-concrete bond and then the post-peak load. The modifications may lead to changes in the ultimate failure mode, concrete cracking model, and apparent CFRP modulus, indicating a potential for a more sustainable design of concrete elements with reduced material usage and maintenance requirements. Further investigation of these factors could offer valuable insights into optimizing the environmental impact of concrete structures.

## 7. Conclusions

This paper presents essential equations to describe the concrete cracking, effective CFRP modulus, and post-peak behavior of 3D CFRP-reinforced concrete. Based on these equations, low-impact concrete beams resulting from optimal non-prismatic geometries can be developed to deliver the desired capacity and pseudo-ductility. The major findings are summarized as follows.

1. The proposed 3D CFRP reinforcements including UC, UCA, UCL, and UCAL are able to improve the tensile contribution of cracked concrete, which can be presented as 0.3$f_{t}$.
2. UC reinforcements can develop considerable load-deflection but tend to fail at a premature compressive strain of 0.0023 or less. The application of aggregate coating UC reinforcement resulted in an enhanced load-deflection stiffness and mitigated premature failure at a concrete compressive strain below 0.003. The implementation of closed loops can effectively mitigate premature failure by compromising the load-deflection stiffness. Based on experimental findings, the apparent modulus of CFRP was adjusted for four different reinforcements to accommodate variations in stiffness.
3. Based on the bond strength and plastic mechanism, the post-peak equations were developed to describe the post-peak capacity and deflections of concrete beams.
4. Based on the calibrated concrete cracking model, the apparent CFRP modulus, and post-peak models, low-impact concrete beams having optimal non-prismatic geometries could be developed to achieve a comparable capacity and ductility to that of a steel-reinforced equivalence.

## Data Availability

The raw/processed data required to reproduce these findings cannot be shared at this time as the data also forms part of an ongoing study.
